# Negative wealth shocks and subsequent depressive symptoms and trajectories in middle-aged and older adults in the USA, England, China, and Mexico: a population-based, multinational, and longitudinal study

**DOI:** 10.1017/S0033291724002332

**Published:** 2024-10

**Authors:** Guangquan Ran, Chuanlong Zuo, Danping Liu

**Affiliations:** 1 Department of Health Related Behaviour and Social Medicine, West China School of Public Health and West China Fourth Hospital, Sichuan University, Chengdu, China; 2 Department of Epidemiology and Biostatistics, West China School of Public Health and West China Fourth Hospital, Sichuan University, Chengdu, China

**Keywords:** negative wealth shocks, depressive symptoms, trajectory, multinational, middle-aged and older adults, cohort study

## Abstract

**Background:**

The association between negative wealth shocks and depression among middle-aged and older individuals remains unclear. Our study aimed to assess the association between negative wealth shocks and depression and its trajectories, and to explore cross-national differences in these associations

**Methods:**

Our sample included 21 999 participants, of which 9519 were from the Health and Retirement Study (2012–2020), 4936 from the English Longitudinal Study of Ageing (2012–2020), 2520 from the China Health and Retirement Longitudinal Study (2011–2020), and 5024 from the Mexican Health and Aging Study (2012–2021). We used latent class trajectory models to identify depressive trajectories, alongside mixed-model logistic regression and multinomial logistic regression to evaluate associations.

**Results:**

In the USA (OR 1.73, 95% CI 1.40–2.16), England (OR 1.71, 95% CI 1.09–2.70), and China (OR 1.38, 95% CI 1.09–1.75), negative wealth shocks were associated with subsequent depressive symptoms, but not in Mexico (OR 1.06, 95% CI 0.86–1.29). Additionally, negative wealth shocks were associated with several depressive trajectories in the USA and China. This association occurred only in increasing–decreasing trajectory in England, while no significant association was found across any trajectory in Mexico.

**Conclusions:**

Negative wealth shocks were associated with subsequent depressive symptoms, with significant associations observed in some specific depressive trajectories. These associations exhibited cross-national differences, underscoring the importance of considering country-specific contexts when addressing the mental health impacts of wealth shocks.

## Introduction

Depression is a major challenge in global mental health, standing out as the primary contributor to the worldwide burden of mental health-related disease (Herrman et al., 2019). Among individuals aged 55 and above, nearly 14% suffer from depression, with an additional 2% suffering from major depression (Kok & Reynolds, 2017). The repercussions of depression extend to severe health, economic, and social impacts, including the high risk of suicide, high medical costs, low quality of life, and damage to human capital (Patel et al., 2016). Despite these ramifications, older individuals with depression are often under-treated (Barry, Abou, Simen, & Gill, 2012), and the effectiveness of antidepressants may be diminished for them (Tedeschini et al., 2011). Therefore, emphasizing prevention is crucial for reducing the burden of late-life depression.

Negative wealth shocks arise from the rapid depletion of assets and/or the accumulation of new debts, leading to an abrupt and substantial net wealth loss. Such shocks can result from macroeconomic events, such as the 2008 Great Recession, or major personal life changes, such as marital breakdown or significant illness (Pool, Needham, Burgard, Elliott, & de Leon, 2017). For middle-aged and older adults who are approaching or already retired, they may fare the worst after negative wealth shocks, as they lack the time to recoup their losses (Butrica, Smith, & Toder, 2010). Previous research indicates that negative wealth shocks are associated with short-term changes in health, including depression (Pool et al., 2017; Swift et al., 2020), psychiatric disorders (Wolfe, Baker, Uddin, & Kirkland, 2022), cognitive decline and dementia (Cho et al., 2023; Pan, Gao, Zhu, & Guo, 2023), and impaired cardiovascular function (Boen & Yang, 2016). However, the research on the association between negative wealth shocks and depression predominantly focused on the population from the USA, lacking evidence from other countries. Many countries around the world are still facing challenges of low economic growth and rising inflation (OECD, 2023), leading to economic fluctuations for numerous households. High-income countries, such as the USA and England, and middle-income countries, such as China and Mexico, face varying levels of economic inequality. When confronted with external shocks like the COVID-19 pandemic, the coping capabilities vary among countries. High-income countries bolstered social protections for the most vulnerable groups, whereas lower-income countries found it a struggle to do so (Nature, 2023). These countries exhibit significant distinctions in both quality and accessibility of social welfare, healthcare resources, and other aspects, which may influence the adverse health outcomes for middle-aged and older adults experiencing negative wealth shocks. Additionally, previous studies have solely used positive depressive symptoms or scores as outcomes, overlooking the dynamic changes in the progression of depression and subgroup differences. Given the complexity of treating depression and the difficulties in diagnosing depression in older patients compared to the younger (Kok & Reynolds, 2017), longitudinal monitoring of depressive symptoms in the elderly is particularly important. And it has a positive impact on the early detection, targeted treatment, and prevention of relapse for depression.

Using harmonized data from population representative, longitudinal studies of aging in the USA, England, China, and Mexico, we assessed the association between negative wealth shocks in middle-aged and older adults and subsequent depressive symptoms and trajectories. We hypothesized that negative wealth shocks are associated with an increase in depressive symptoms and that this association exhibits cross-national differences. Additionally, we proposed that the impact of negative wealth shocks on depressive symptoms differed across specific depressive trajectories within different countries. Our study aimed to explore these associations and the potential differences across the four countries.

## Methods

### Study design and participants

We used the datasets of Health and Retirement Study (HRS, waves 11–15 [2012–2020]) (Sonnega et al., 2014) in the United States and related aging surveys: the English Longitudinal Study of Ageing (ELSA, waves 6–10 [2012–2021]) (Steptoe, Breeze, Banks, & Nazroo, 2013), the China Health and Retirement Longitudinal Study (CHARLS, waves 1–5 [2011–2020]) (Zhao, Hu, Smith, Strauss, & Yang, 2014), and the Mexican Health and Aging Study (MHAS, waves 3–6 [2012–2021]) (Wong, Michaels-Obregon, & Palloni, 2017), which are population-based, prospective cohort studies. We selected the waves above primarily based on the consideration of timeliness and cross-country comparability of the data. The minimum entry age is 50 in the HRS, ELSA, and MHAS, and 45 in the CHARLS. The process of participant selection is shown in online Supplementary Figs S1–S4.

All four cohort studies underwent ethical committee reviews in their respective countries, and all participants provided written informed consent. HRS received approval from the Health Sciences/Behavioural Sciences Institutional Review Board at the University of Michigan. ELSA was approved by NHS Research Ethics Committees under the National Research and Ethics Service. CHARLS obtained approval from the Institutional Review Board at Peking University. MHAS was approved by the ethics committee of the University of Texas Medical Centre.

### Measurement of negative wealth shocks

In each country, household wealth was measured at the couple level. We computed negative wealth shocks using data from 2012 to 2014 for the HRS and the ELSA, from 2011 to 2013 for the CHARLS, and from 2012 to 2015 for the MHAS. The wealth variables are sourced from the RAND HRS Longitudinal File and harmonized ELSA, CHARLS, and MHAS datasets available on the Gateway to Global Aging Data website. The components of wealth variables for each country are shown in online Supplementary Table S1.

To ensure comparability, we adjusted all net wealth to the 2014 levels using the consumer price index within each country. Per capita wealth in each wave was calculated by dividing total family wealth for couples by 1.5 and for single households by 1. Consistent with previous research, negative wealth shocks were defined as a net wealth loss of 75% or more between two consecutive waves (Pool et al., 2017; Pool et al., 2018).

### Measurement of depression

All cohorts utilized the Centre for Epidemiologic Studies Depression Scale (CES-D) to assess depressive symptoms (Radloff, 1977), but the items and scoring methodologies differed. HRS and ELSA comprised eight items, with a maximum score of 8. A score of 3 or higher was defined as positive for depressive symptoms (Turvey, Wallace, & Herzog, 1999). CHARLS comprised 10 items, with a maximum score of 30. A score of 10 or higher was defined as positive for depressive symptoms (Fu, Si, & Guo, 2022). MHAS comprised nine items, with a maximum score of 9. A score of 5 or higher was defined as positive for depressive symptoms (Aguilar-Navarro, Fuentes-Cantú, Avila-Funes, & García-Mayo, 2007).

Depression was measured at three to four different time points across the countries. Specifically, in the HRS, depression was collected in 2014, 2016, 2018, and 2020; in the ELSA, depression was collected in 2014, 2016, 2018, and 2021; in the CHARLS, depression was collected in 2013, 2015, 2018, and 2020; and in the MHAS, depression was collected in 2015, 2018, 2018, and 2021. To avoid reverse causation, the first time point of depression measurement was excluded as an outcome indicator when assessing the association between negative wealth shocks and subsequent depressive symptoms. When fitting depression trajectories, data from all depression measurement time points was used.

**Figure 1.**
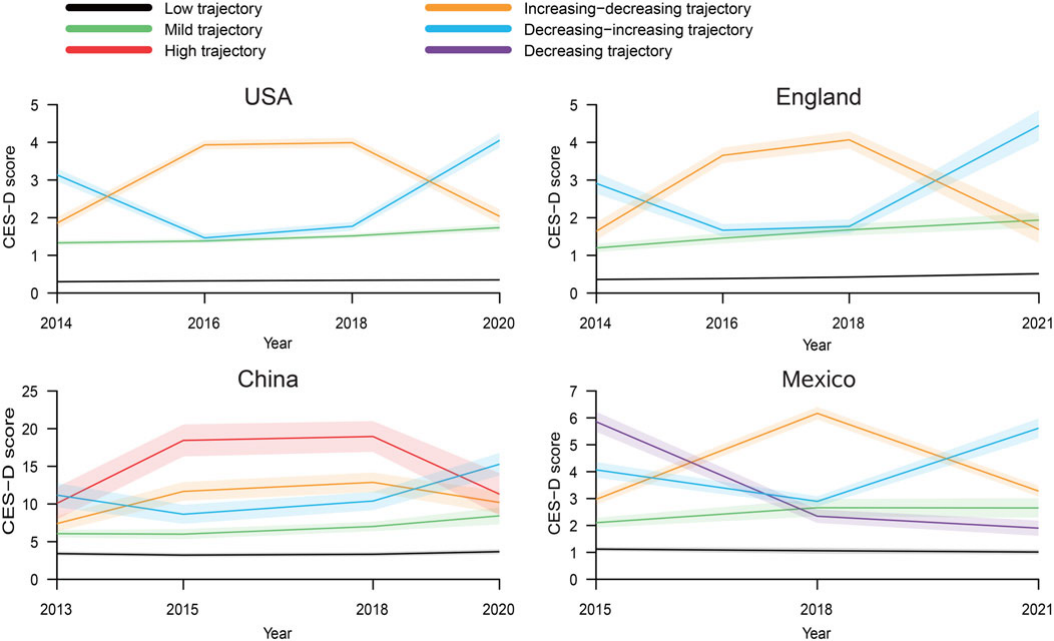
Trajectories of depressive symptoms after baseline, by country.

The six distinct depression trajectories we identified were as follows: (1) symptoms maintaining low CES-D scores throughout the entire follow-up period (low); (2) having moderate depressive symptoms throughout the follow-up period with CES-D scores below the threshold (mild); (3) experiencing an initial increase and subsequent decrease in CES-D scores during the follow-up period (increasing–decreasing); (4) experiencing an initial decrease and subsequent increase in CES-D scores during the follow-up period (decreasing–increasing); (5) maintaining high CES-D scores throughout the entire follow-up period (high); and (6) experiencing a decrease in CES-D scores over the follow-up period (decreasing). Ultimately, individuals in HRS and ELSA were divided into four trajectories, while those in CHARLS and MHAS were divided into five trajectories (Fig. 1). The baseline characteristics according to the trajectories of depressive symptoms are shown in the online Supplementary Tables S6–S9.

### Covariates

Considering established risk factors associated with wealth shocks and depression from previous research (Pan et al., 2023; Pool et al., 2017, 2018; Swift et al., 2020), the following baseline covariates were considered for inclusion: age, gender (male or female), education (lower secondary education or below, upper secondary, and higher than upper secondary), minority group (minority racial group for HRS and ELSA, and rural or urban residential status for CHARLS and MHAS), household size, alcohol use (none, moderate [⩽2 drinks per day], and heavy [＞2 drinks per day]), smoking status (never smoked, former smoker, and current smoker), body mass index (BMI; calculated as weight in kilograms divided by height in meters squared), and total wealth per capita (adjusted to 2014 US dollars using consumer price index and exchange rates). Additionally, we considered several time-varying covariates in the analysis, including marital status (married or living with partner, separated or divorced, widowed, and never married), self-report of health status (excellent, very good, good, fair, and poor), limitations in any of five activities of daily living (ADLs; limitations in any of 5 ADLs: dressing, bathing, eating, getting in/out of bed, and using the toilet), and number of self-reported diagnosed health problems (0, 1, 2, and ⩾3 for each of hypertension, diabetes, cancer, stroke, arthritis, heart problems, and chronic lung disease).

### Statistical analysis

We utilized latent class trajectory models to identify trajectories of depressive symptoms over time. This is a model capable of simplifying heterogeneous populations into more homogeneous clusters or classes, thereby potentially incorporating random effects to allow individual variations within these classes (Lennon et al., 2018). The determination of the optimal number of trajectories was based on the minimum Bayesian information criterion, considering posterior probability (>0.7) and class size (>2%) for each class (Mirza et al., 2016).

To address potential confounding due to time-varying changes in some covariates, we applied inverse probability of treatment weights (IPTW) (Cole & Hernán, 2008). This method involved calculating the probability of each participant experiencing a negative wealth shock based on observed covariates, and then using these probabilities to create a weighted sample in which the distribution of covariates is independent of the exposure. The weights were derived from a logistic regression model that included baseline and time-varying covariates. The application of IPTW mitigated confounding bias by balancing covariates across exposure groups to obtain unbiased estimates of the association between negative wealth shocks and depression.

We used mixed-model logistic regression to estimate the association between negative wealth shocks and subsequent positive for depressive symptoms, in which a participant-specific random intercept was applied. Initially, separate analyses were conducted to estimate the association within each country. Subsequently, we performed a pooled analysis by combining data from all countries. In the pooled analysis, an interaction term between negative wealth shocks and country was included to estimate the marginal differences for England, China, and Mexico relative to the USA as the reference. Furthermore, we used multinomial logistic regression to assess the association between negative wealth shocks and trajectories of depressive symptoms for each country. All models were adjusted for the aforementioned covariates. To address the missing covariate data, we performed multiple imputations for all missing covariates.

We performed several additional analyses to examine the robustness of our results: (1) we conducted subgroup analyses for participants aged 65 years and older *v.* those younger than 65 years; (2) we conducted subgroup analyses based on gender; (3) we performed analyses on participants with complete covariate data; (4) we excluded participants who did not experience negative wealth shocks in the second wave but did so in subsequent waves; (5) we conducted analyses controlling for labor force participation and pension receipt as time-varying variables; (6) to eliminate the potential impact of COVID-19, we excluded data collected after 2019 and reanalyzed the results; (7) we used a 50% threshold for defining a negative wealth shock instead of 75%.

Our study followed the Strengthening the Reporting of Observational Studies in Epidemiology (STROBE) reporting guideline. Statistical significance was defined as *p* < 0.05 and all *p* values were two-sided. All statistical analyses were performed using R version 4.4.0.

## Results

The study population comprised 21 999 participants from four countries (Table 1). The mean age varied across countries, with China having the youngest average age (56.8 years) and the USA, England, and Mexico having older populations (65.3, 65.5, and 62.4 years, respectively). Gender distribution showed a higher proportion of males in China (54.3%). The vast majority of participants had only lower secondary education or below in China (87.1%) and Mexico (83.6%), whereas participants in the USA and England had higher levels of education. Household size and wealth per capita also differed notably. The median household size was larger in Mexico (4 people) and China (3 people), compared to two people in the USA and England. Wealth per capita was highest in England (median $354 049.4) and lowest in China (median $8631.2). The incidence of negative wealth shocks in the USA (7.5%) and England (3.2%) were lower than that in China (13.9%) and Mexico (15.4%). Characteristics of excluded participants were shown in the online Supplementary Tables S2–S5.

**Table 1.** Characteristics of participant, by country

	HRS (USA, *n* = 9519)	ELSA (England, *n* = 4936)	CHARLS (China, *n* = 2520)	MHAS (Mexico, *n* = 5024)
Mean age(years)	65.3(9.2)	65.5(8.3)	56.8(7.9)	62.4(7.8)
Gender				
Male	4034(42.4)	2286(46.3)	1369(54.3)	2340(46.6)
Female	5485(57.6)	2650(53.7)	1151(45.7)	2684(53.4)
Education				
Lower secondary education or below	1246(13.1)	1104(22.4)	2194(87.1)	4199(83.6)
Upper secondary	1099(32.6)	2668(54.1)	294(11.7)	201(4.0)
Higher than upper secondary	5174(54.4)	1164(23.6)	32(1.3)	624(12.4)
Marital status				
Married or living with partner	6674(70.1)	3767(76.3)	2330(92.5)	3840(76.4)
Separated or divorced	1231(12.9)	452(9.2)	21(0.8)	349(6.9)
Widowed	1183(12.4)	477(9.7)	154(6.1)	604(12.0)
Never married	431(4.5)	240(4.9)	15(0.6)	231(4.6)
Minority group	2527(26.5)	133(2.7)	1645(65.3)	1569(31.2)
Median household size (people)	2(2–3)	2(2–2)	3(2–5)	4(3–6)
Wealth per capital (2014 US dollars)	134 049.8(29 834.7–384 276.0)	354 049.4(198 609.0–589 752.7)	8631.2(2718.8–19 182.4)	32 826.4(12 490.9–72 467.0)
Alcohol use				
None	5409(56.8)	1527(39.9)	1642(65.1)	4016(79.9)
Moderate	3199(33.6)	2020(40.9)	835(33.1)	481(9.6)
Heavy > 2	911(9.6)	1389(28.1)	43(1.7)	527(10.5)
Smoking status				
Never smoked	4449(46.7)	2036(41.3)	1461(58.0)	3103(61.8)
Former smoker	3968(41.7)	2453(49.7)	179(7.1)	1282(25.5)
Current smoker	1102(11.6)	447(9.1)	880(34.9)	639(12.7)
BMI	28.7(5.8)	28.2(4.9)	23.9(3.7)	27.7(4.5)
Self-report of health status				
Excellent	188(2.0)	103(2.1)	34(1.4)	242(4.8)
Very good	1278(13.4)	668(13.5)	350(13.9)	2449(48.8)
Good	3189(33.5)	1636(33.1)	1362(54.0)	1895(37.7)
Fair	3656(38.4)	1747(35.4)	552(21.9)	284(5.7)
Poor	1208(12.7)	782(15.8)	222(8.8)	154(3.1)
ADL limitation	708(7.4)	494(10.0)	170(6.7)	342(6.8)
Number of self-reported diagnosed health problems				
0	1862(19.6)	1649(33.4)	1273(50.5)	1986(39.5)
1	2776(29.2)	1725(34.9)	821(32.6)	1766(35.2)
2	2555(26.8)	1072(21.7)	317(12.6)	950(18.9)
⩾3	2326(24.4)	490(9.9)	109(4.3)	322(6.4)
Negative wealth shocks	711(7.5)	156(3.2)	351(13.9)	775(15.4)

BMI, body mass index, calculated as weight in kilograms divided by height in meters squared; ADL, activities of daily living; IQR, interquartile range.

Data are mean (s.d.), *n* (%), or median (IQR).

**Figure 2.**
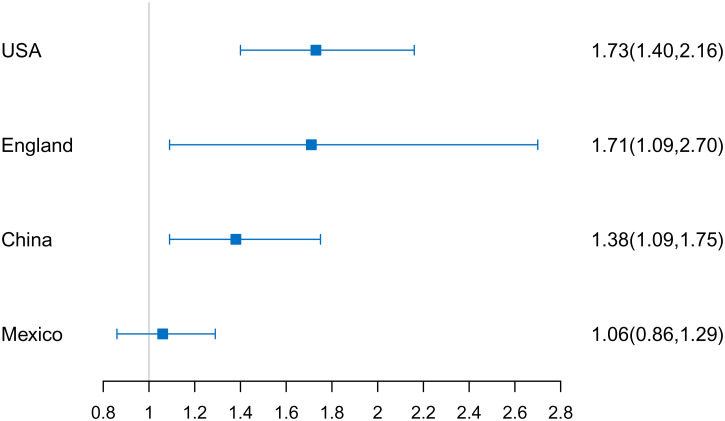
Associations of negative wealth shocks with subsequent positive for depressive symptoms.

In the follow-up after the second wave, the proportion of individuals with positive for depressive symptoms at one or more episodes was the highest in China (48.3%), followed by Mexico (30.5%), the USA (24.3%) and England (23.6%). In the USA (OR 1.73; 95% CI 1.40–2.16), England (OR 1.71; 95% CI 1.09–2.70), and China (OR 1.38; 95% CI 1.09–1.75), negative wealth shocks were associated with subsequent positive for depressive symptoms, but this was not the case in Mexico (OR 1.06; 95% CI 0.86–1.29) (Fig. 2). Additionally, the results of the interaction between negative wealth shocks and country variable in the pooled model indicated that the estimate for Mexico (*p* = 0.01) was significantly different from that for the USA, whereas that was not for England (*p* = 0.56) or China (*p* = 0.50).

Furthermore, using the low trajectory group as a reference, we assessed the impact of negative wealth shocks on different depression trajectories within four countries. In the USA, negative wealth shocks were associated with all three other trajectories. The highest risk occurred in the increasing–decreasing trajectory (OR 2.01; 95% CI 1.54–2.64), followed by decreasing–increasing (OR 1.47; 95% CI 1.13–1.93) and mild (OR 1.40; 95% CI 1.12–1.74) trajectories. In England, negative wealth shocks were only associated with the increasing–decreasing trajectory (OR 2.05; 95% CI 1.09–3.87). In China, this association occurred in the high trajectory (OR 2.41; 95% CI 1.25–4.64), increasing–decreasing (OR 1.57; 95% CI 1.09–2.24), and mild (OR 1.51; 95% CI 1.15–1.99) trajectories. Conversely, in Mexico, such an association was not found in any trajectory (Table 2).

**Table 2:** Associations of negative wealth shocks with trajectories of depressive symptoms, by country

	Trajectory	Participants, No.	Odds Ratio (95% CI)
HRS (the USA)	Low	6398	Reference
	Mild	1517	1.40(1.12-1.74)
	Increasing-decreasing	698	2.01(1.54-2.64)
	Decreasing-increasing	906	1.47(1.13-1.93)
ELSA(England)	Low	3475	Reference
	Mild	772	1.19(0.71-1.99)
	Increasing-decreasing	280	2.05(1.09-3.87)
	Decreasing-increasing	409	1.49(0.81-2.74)
CHARLS(China)	Low	1149	Reference
	Mild	733	1.51(1.15-1.99)
	Increasing-decreasing	310	1.57(1.09-2.24)
	Decreasing-increasing	271	1.39(0.94-2.06)
	High	57	2.41(1.25-4.64)
MHAS(Mexico)	Low	2175	Reference
	Mild	916	1.15(0.94-1.42)
	Increasing-decreasing	742	1.01(0.80-1.27)
	Decreasing-increasing	879	1.19(0.96-1.47)
	Decreasing	312	1.25(0.92-1.71)

Adjusted for age, gender, education, marital status, minority group, household size, wealth per capital, alcohol use, smoking status, BMI, self-report of health status, ADL limitation, and number of self-reported diagnosed health problems

Subgroup analyses by age revealed stronger associations in participants younger than 65 years across all countries. The sensitivity analysis results were largely consistent with the main analysis, despite a potential decrease in statistical power and some differences in the fitting and analysis of depressive trajectories due to the reduced sample size (online Supplementary Table S10). Analyses limited to individuals with complete covariate data confirmed the robustness of the main findings, with similar odds ratios observed (online Supplementary Fig. S5 and Table S11). Excluding individuals who did not experience negative wealth shocks in the second wave but did so afterwards also yielded consistent results (online Supplementary Fig. S6 and Table S12). Additional adjustments for labor force participation and pension receipt, both separately and combined, did not substantially alter the results (online Supplementary Tables S13–S15). When excluding data after 2019 to account for COVID-19, the associations remained robust. Finally, using a 50% threshold for negative wealth shocks also yielded similar patterns, with different reductions in odds ratio in the four countries (online Supplementary Table S16).

## Discussion

In this large-scale, population representative, multinational, and longitudinal study, we found that negative wealth shocks encountered by the middle-aged and older adults in the USA, England, and China were associated with subsequent positive for depressive symptoms, but this association was not observed in Mexico. Notably, this study represents the first of its kind to establish a connection between negative wealth shocks and trajectories of depressive symptoms, enabling the tracking of their longitudinal dynamic changes. In the USA and China, negative wealth shocks were associated with several depressive trajectories, indicating varying levels of impact on mental health. In England, this association mainly occurred in the increasing–decreasing trajectory, while no significant association was found across any trajectory in Mexico.

Previous relevant research has predominantly focused on the impact of wealth shocks on the population in the USA, possibly influenced by the Great Recession. However, this may leave other countries, especially middle-income and low-income countries, relatively neglected. Our study explores further and the results are generally consistent with these studies. A study utilizing the HRS (*n* = 19 281) assessed the association between negative wealth shocks and depression among late middle-aged adults in the USA (Pool et al., 2017). It was consistent with our results. Moreover, within the USA, income decline, increased debt (Swift et al., 2020), and diminished housing wealth are associated with an increase in depressive symptoms (Yilmazer, Babiarz, & Liu, 2015). In England and China, household wealth is significantly associated with depressive symptoms (Qin, Evandrou, Falkingham, & Vlachantoni, 2022; Zhang, Zhang, Xia, Feng, & Wu, 2022). However, the association between economic conditions and depression appears to be more complex in Mexico (Anand, Esposito, & Villaseñor, 2018). These findings suggest the impact of negative wealth shocks on health among middle-aged and older adults may be influenced by government policies and societal backgrounds.

In trajectory classification, participants from two high-income countries were divided into four trajectories, while those from two middle-income countries were divided into five trajectories, including an additional ‘high trajectory’ in China and ‘decreasing trajectory’ in Mexico. This may be the result of differences in depressive symptoms due to inequalities at the national level, including disparities in economics, healthcare support, and social structures. Using the low trajectory as the reference, negative wealth shocks in the USA were high-risk factors for all trajectories, indicating a broad impact on depressive symptoms following financial distress. In England, negative wealth shocks were only associated with a high risk in the increasing–decreasing trajectory. This may suggest that the pattern of depressive symptoms over time in response to wealth shocks is more complex and possibly more transient. In China, the associations were observed in mild, increasing–decreasing, and high trajectories, reflecting a diverse response to financial adversity, including a severe impact on a subset of the population with persistent high depressive symptoms. While in Mexico, there was no association in any trajectory, suggesting a different socio-economic resilience or social safety nets that may buffer against the mental health impacts of financial shocks. One possible explanation is the non-contributory social pension program for citizens aged 70 and above in Mexico, which likely played a role in reducing income inequality and alleviating the impact of wealth shocks among older adults, consequently influencing mental well-being (Salinas-Rodriguez, Torres-Pereda Mdel, Manrique-Espinoza, Moreno-Tamayo, & Tellez-Rojo Solis, 2014). These findings highlight the importance of considering country-specific contexts and the nature of depressive trajectories when evaluating the mental health impact of financial shocks. The variations observed may be influenced by differences in social protection systems, cultural attitudes toward financial stress, and the availability of mental health resources.

Our findings substantiate the pathway linking wealth shocks and stress response. Stressful life events are established risk factors for depression (Kendler, Karkowski, & Prescott, 1999), as stress can trigger neuronal microdamage and neuroinflammatory activation in the brain, and that inflammatory mediators can induce depressive symptoms (Wager-Smith & Markou, 2011). Negative wealth shocks in middle and older age are a stressful life event, given the reduction in income-earning potential. Weaker economic recovery capabilities after the shocks may lead to permanent changes in their economic status (Butrica et al., 2010). The trajectory analysis results indicate that the odds of high depressive symptoms were higher among individuals exposed to negative wealth shocks compared to the reference trajectory with low depressive symptoms. Moreover, negative wealth shocks in the USA, England, and China are all associated with the increasing–decreasing trajectory. Over the subsequent 2–5 years, the impact of negative wealth shocks gradually intensifies, after which depressive levels start to decline. The emergence of this decrease may provide an intervenable perspective, and further research into what triggers the decrease in depression holds public health significance.

Our results indicated that the association between negative wealth shocks and depressive symptoms was stronger in participants under the age of 65 across all countries. Late middle-aged adults often face significant financial responsibilities, such as supporting children and aging parents (Infurna, Gerstorf, & Lachman, 2020; Riitsalu, Sulg, Lindal, Remmik, & Vain, 2023), and may have less stable economic conditions compared to older adults who might benefit from more secure financial arrangements or social support systems (Litwin & Sapir, 2009). And older adults, with higher levels of experience-based knowledge and lower levels of negative emotions, have better financial decision-making (Eberhardt, de Bruin, & Strough, 2019). As a result, the late middle-aged adults might be particularly vulnerable to wealth shocks.

This study has several limitations. Firstly, estimates might be biased due to residual confounding. It must be noted that the selection of covariates is constrained by the availability in the four databases. For the pooling and comparison of models, on one hand, we only chose covariates that were common across the four cohorts; on the other hand, we coarsened the categorization of certain covariates. This may limit the depth of interpretation of our results. Secondly, Mexico had one fewer wave than the other three countries due to data limitations. This could introduce bias in the estimation of its long-term trajectories and cross-national comparisons. Thirdly, the number of items and scoring rules for the CES-D scale varied across countries, which could impact the fitting of depression trajectories. Fourthly, there was a potential for reverse causality, where depressive symptoms might lead to wealth loss. Although we excluded individuals with baseline depression, this approach did not fully control for pre-existing depression prior to the wealth shocks. Fifthly, changes in wealth following the wealth shocks were ignored, which may influence our results. Sixthly, there was some selection bias in this study, as excluded individuals may differ from included individuals in terms of negative wealth shocks and depressive symptoms.

The strength of this study lies in its utilization of large sample data from four different middle-income and high-income countries. We compared the potential effects of negative wealth shocks on depressive symptoms in middle-aged and older adults across different countries, taking into account the longitudinal dynamic development of depressive symptoms.

Our study results elucidate the impact of negative wealth shocks on depression from the perspective of countries with different income levels, which is crucial for addressing wealth inequality and providing healthcare support for individuals with depression. In a global perspective, the role of wealth inequality in adverse health outcomes may be related to differences in policies, social welfare, and healthcare support among countries. Consequently, it is important for countries to allocate resources reasonably, reduce wealth disparities, and maintain economic stability to alleviate depressive symptoms in the middle-aged and older adults resulting from negative wealth shocks. Further prospective and intervention studies are necessary to validate our findings and explore the mechanisms and reasons underlying these cross-national differences in this association.

## Supporting information

Ran et al. supplementary material Ran et al. supplementary material

## Supplementary material

The supplementary material for this article can be found at https://doi.org/10.1017/S0033291724002332
