# Supplementary material for: Negative wealth shocks and subsequent depressive symptoms and trajectories in middle-aged and older adults in the USA, England, China, and Mexico: a population-based, multinational, and longitudinal study
Source: Psychol Med. 2024 Oct 14;54(14):3888–95. doi: 10.1017/S0033291724002332 (PMC11578904; doi:10.1017/S0033291724002332)
Supplement: Ran et al. supplementary material [file S0033291724002332sup001.docx]

**Supplementary Material**

**Supplementary Figure 1.** The flowchart for the process of participant selection in the USA

**Supplementary Figure 2.** The flowchart for the process of participant selection in England

**Supplementary Figure 3.** The flowchart for the process of participant selection in China

**Supplementary Figure 4.** The flowchart for the process of participant selection in Mexico

**Supplementary Table 1.** The components of wealth variables, by country

**Supplementary Table 2.** Comparison of characteristics between the included and excluded participants in the USA

**Supplementary Table 3.** Comparison of characteristics between the included and excluded participants in England

**Supplementary Table 4.** Comparison of characteristics between the included and excluded participants in China

**Supplementary Table 5.** Comparison of characteristics between the included and excluded participants in Mexico

**Supplementary Table 6.** Characteristics of the participants in the USA according to the trajectories of depressive symptoms

**Supplementary Table 7.** Characteristics of the participants in England according to the trajectories of depressive symptoms

**Supplementary Table 8.** Characteristics of the participants in China according to the trajectories of depressive symptoms

**Supplementary Table 9.** Characteristics of the participants in Mexico according to the trajectories of depressive symptoms

**Supplementary Table 10.** Associations of negative wealth shocks with subsequent positive for depressive symptoms—results of additional analysis

**Supplementary Figure 5.** Trajectories of depressive symptoms after baseline—only individuals included with complete covariates, by country

**Supplementary Table 11.** Associations of negative wealth shocks with trajectories of depressive symptoms —only individuals included with complete covariates, by country

**Supplementary Figure 6.** Trajectories of depressive symptoms after baseline—excluding individuals who did not experience negative wealth shocks in the second wave but did so afterwards, by country

**Supplementary Table 12.** Associations of negative wealth shocks with trajectories of depressive symptoms —excluding individuals who did not experience negative wealth shocks in the second wave but did so afterwards, by country

**Supplementary Table 13.** Associations of negative wealth shocks with trajectories of depressive symptoms —additional adjustment for labor force participation, by country

**Supplementary Table 14.** Associations of negative wealth shocks with trajectories of depressive symptoms —additional adjustment for pension receipt, by country

**Supplementary Table 15.** Associations of negative wealth shocks with trajectories of depressive symptoms —additional adjustment for labor force participation and pension receipt, by country

**Supplementary Table 16.** Associations of negative wealth shocks with trajectories of depressive symptoms —50% threshold for negative wealth shocks, by country

**Supplementary Figure 1. The flowchart for the process of participant selection in the USA**


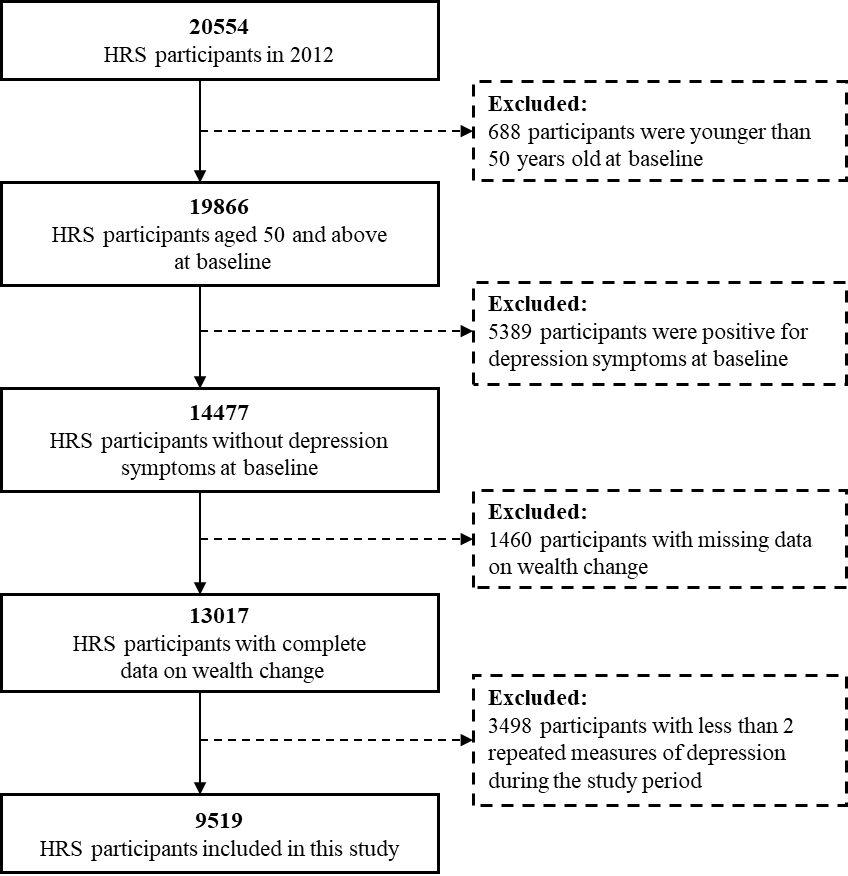


**Supplementary Figure 2. The flowchart for the process of participant selection in England**


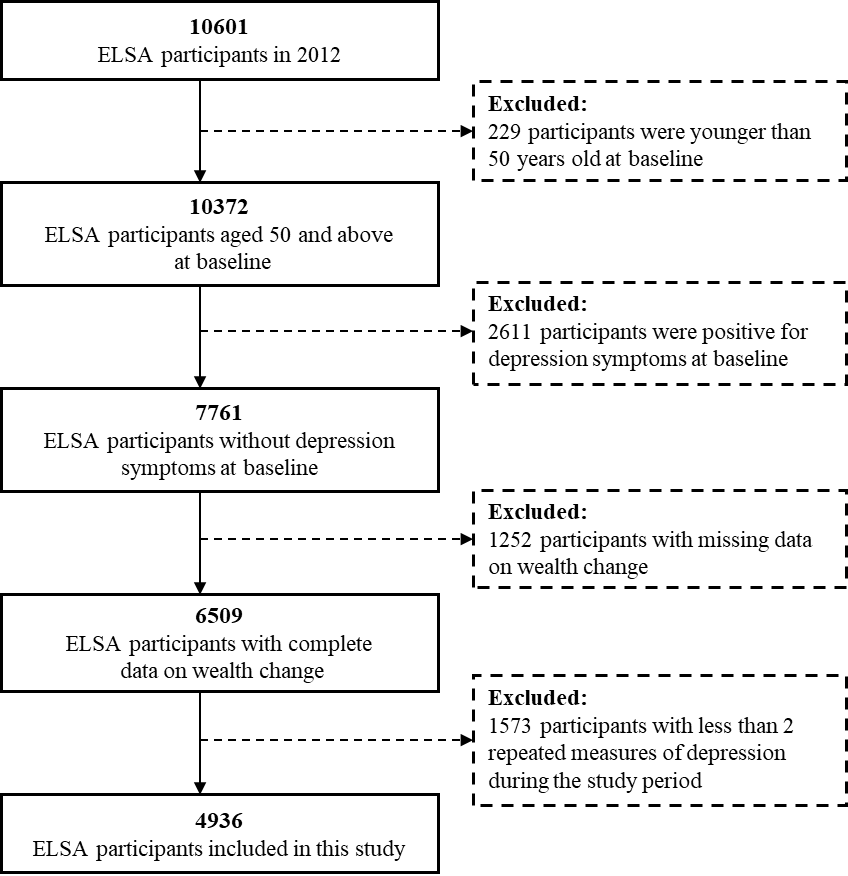


**Supplementary Figure 3. The flowchart for the process of participant selection in China**


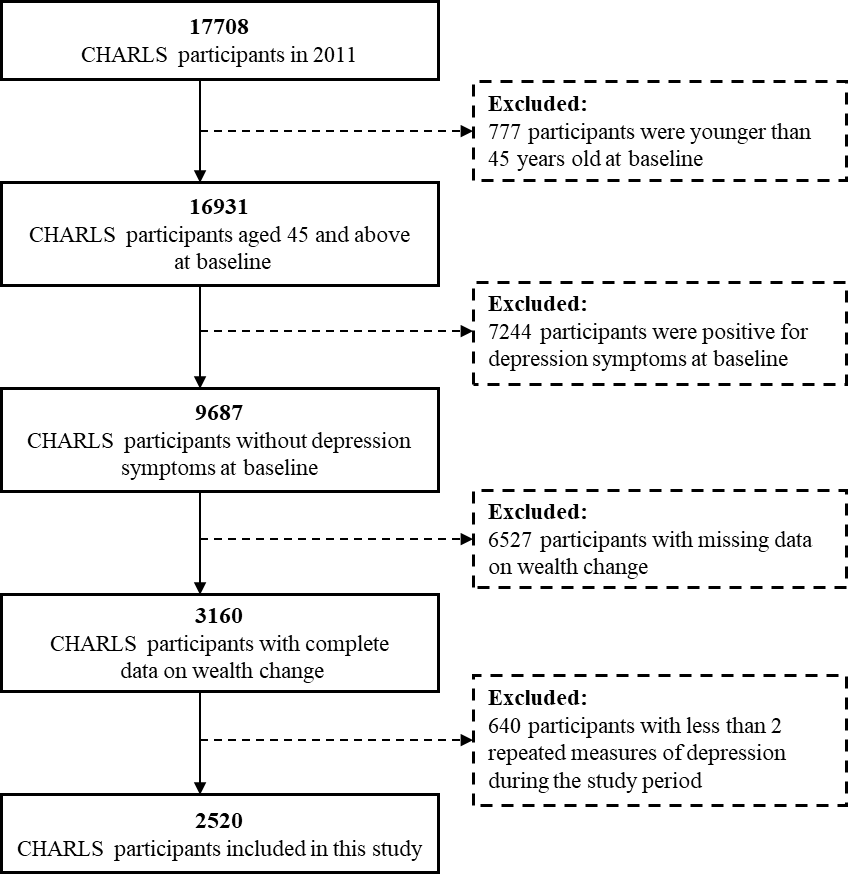


**Supplementary Figure 4. The flowchart for the process of participant selection in Mexico**


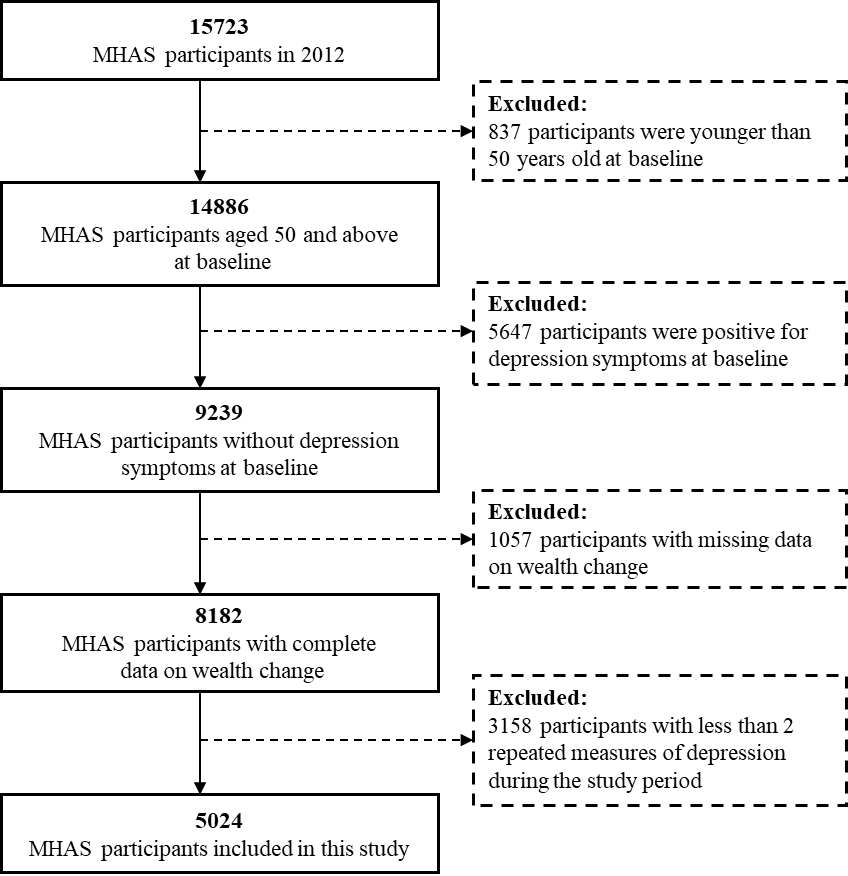


**Supplementary Table 1. The components of wealth variables, by country**

| **Components** | **The United States** | **England** | **China** | **Mexico** |
| --- | --- | --- | --- | --- |
| Primary residence and other real estate | √ | √ | √^b^ | √ |
| Transportation | √ |  | √^c^ | √ |
| Business | √ | √ | √^d^ | √ |
| Non-housing financial wealth^a^ | √ | √ | √ | √ |
| Debts | √ | √ | √ | √ |

^a^ Non-housing financial wealth includes stocks, mutual funds, investment trusts, checking, savings, and money market accounts, CD, government savings bonds, T-bills, bonds, bond funds and all other savings

^b^ The value of primary residence with the percentage of ownership are considered

^c^ The total value of transportation vehicles is measured at the household level at wave 1 and 2.

^d^ The total value of non-financial assets, fixed capital assets, irrigable land, and all livestock and aquatic life is included and measured at the household level at wave 1 and 2.

**Supplementary Table 2. Comparison of characteristics between the included and excluded participants in the USA**

|  | Included(n=9519) | Excluded(n=4958) | *P* value |
| --- | --- | --- | --- |
| Mean age(years) | 65.5 (8.3) | 68.9 (10.7) | <0.001 |
| Gender |  |  | <0.001 |
| Male | 4034(42.4) | 2338(47.2) |  |
| Female | 5485(57.6) | 2620(52.8) |  |
| Education |  |  | <0.001 |
| Lower secondary education or below | 1246(13.1) | 948(19.1) |  |
| Upper secondary | 3098(32.6) | 1758(35.5) |  |
| Higher than upper secondary | 5173(54.4) | 2251(45.4) |  |
| Marital status |  |  | <0.001 |
| Married or living with partner | 6674(70.1) | 3053(61.7) |  |
| Separated or divorced | 1231(12.9) | 614(12.4) |  |
| Widowed | 1183(12.4) | 1067(21.6) |  |
| Never married | 431(4.5) | 213(4.3) |  |
| Minority group | 2515(26.5) | 1186(24.0) | 0.001 |
| Median household size  (people) | 2(2 to 3) | 2(1 to 2) | <0.001 |
| Wealth per capital (2014 US dollars) | 134049.8(29869.0 to 384201.1) | 120644.8(25881.9 to 339609.9) | 0.001 |
| Alcohol use |  |  | <0.001 |
| None | 5396(56.8) | 3207(65.0) |  |
| Moderate | 3192(33.6) | 1334(27.0) |  |
| Heavy | 909(9.6) | 395(8.0) |  |
| Smoking status |  |  | <0.001 |
| Never smoked | 4430(46.7) | 2091(42.5) |  |
| Former smoker | 3952(41.7) | 2135(43.4) |  |
| Current smoker | 1098(11.6) | 691(14.1) |  |
| BMI | 28.7(5.8) | 27.6(5.9) | <0.001 |
| Self-report of Health Status |  |  | <0.001 |
| Excellent | 188(2.0) | 311(6.3) |  |
| Very good | 1276(13.4) | 1047(21.1) |  |
| Good | 3187(33.5) | 1672(33.8) |  |
| Fair | 3656(38.4) | 1487(30.0) |  |
| Poor | 1208(12.7) | 436(8.8) |  |
| ADL limitation | 705(7.4) | 717(14.6) | <0.001 |
| Number of self-reported diagnosed health problems |  |  | <0.001 |
| 0 | 1862(19.6) | 684(13.8) |  |
| 1 | 2776(29.2) | 1096(22.1) |  |
| 2 | 2555(26.8) | 1279(25.8) |  |
| ≥3 | 2326(24.4) | 1899(38.3) |  |
| Negative wealth shocks | 711(7.5) | 349(10.0) | <0.001 |

Data are mean (SD), n (%), or median (IQR).

Abbreviations: BMI, body mass index, calculated as weight in kilograms divided by height in meters squared; ADL, activities of daily living; IQR, interquartile range.

**Supplementary Table 3. Comparison of characteristics between the included and excluded participants in England**

|  | Included(n=4936) | Excluded(n=2825) | *P* value |
| --- | --- | --- | --- |
| Mean age(years) | 65.5(8.3) | 68.9(10.7) | <0.001 |
| Gender |  |  | 0.119 |
| Male | 2286(46.3) | 1361(48.2) |  |
| Female | 2650(53.7) | 1464(51.8) |  |
| Education |  |  | <0.001 |
| Lower secondary education or below | 998(21.9) | 910(34.9) |  |
| Upper secondary | 2470(54.1) | 1243(47.6) |  |
| Higher than upper secondary | 1098(24.0) | 457(17.5) |  |
| Marital status |  |  | <0.001 |
| Married or living with partner | 3767(76.3) | 1993(70.6) |  |
| Separated or divorced | 452(9.2) | 221(7.8) |  |
| Widowed | 477(9.7) | 476(16.9) |  |
| Never married | 240(4.9) | 133(4.7) |  |
| Minority group | 133(2.7) | 109(3.9) | 0.006 |
| Median household size  (people) | 2(2 to 2) | 2(2 to 2) | 0.002 |
| Wealth per capital (2014 US dollars) | 354049.4(198609.0 to 589752.7) | 277344.3(144659.2 to 477401.0) | <0.001 |
| Alcohol use |  |  | <0.001 |
| None | 1399(30.7) | 902(37.9) |  |
| Moderate | 1874(41.1) | 879(36.9) |  |
| Heavy | 1289(28.3) | 601(25.2) |  |
| Smoking status |  |  | <0.001 |
| Never smoked | 2036(41.3) | 1003(35.5) |  |
| Former smoker | 2452(49.7) | 1464(51.8) |  |
| Current smoker | 447(9.1) | 357(12.6) |  |
| BMI | 28.2(4.9) | 27.9(5.1) | 0.037 |
| Self-report of Health Status |  |  | <0.001 |
| Excellent | 103(2.1) | 170(6.0) |  |
| Very good | 668(13.5) | 583(20.7) |  |
| Good | 1636(33.1) | 933(33.1) |  |
| Fair | 1747(35.4) | 815(28.9) |  |
| Poor | 782(15.8) | 321(11.4) |  |
| ADL limitation | 493(10.0) | 434(15.4) | <0.001 |
| Number of self-reported diagnosed health problems |  |  | <0.001 |
| 0 | 1649(33.4) | 796(28.2) |  |
| 1 | 1725(34.9) | 905(32.0) |  |
| 2 | 1072(21.7) | 633(22.4) |  |
| ≥3 | 490(9.9) | 490(17.4) |  |
| Negative wealth shocks | 156(3.2) | 83(5.3) | <0.001 |

Data are mean (SD), n (%), or median (IQR).

Abbreviations: BMI, body mass index, calculated as weight in kilograms divided by height in meters squared; ADL, activities of daily living; IQR, interquartile range.

**Supplementary Table 4. Comparison of characteristics between the included and excluded participants in China**

|  | Included(n=2520) | Excluded(n=7167) | *P* value |
| --- | --- | --- | --- |
| Mean age(years) | 56.8(7.9) | 59.0(9.9) | <0.001 |
| Gender |  |  | 0.097 |
| Male | 1369(54.3) | 3754(52.4) |  |
| Female | 1151(45.7) | 3413(47.6) |  |
| Education |  |  | <0.001 |
| Lower secondary education or below | 2194(87.1) | 6001(83.7) |  |
| Upper secondary | 294(11.7) | 928(12.9) |  |
| Higher than upper secondary | 32(1.3) | 238(3.3) |  |
| Marital status |  |  | <0.001 |
| Married or living with partner | 2330(92.5) | 6374(88.9) |  |
| Separated or divorced | 21(0.8) | 77(1.1) |  |
| Widowed | 154(6.1) | 668(9.3) |  |
| Never married | 15(0.6) | 48(0.7) |  |
| Minority group | 1645(65.3) | 3701(51.6) | <0.001 |
| Median household size  (people) | 3(2 to 5) | 3(2 to 5) | 0.001 |
| Wealth per capital (2014 US dollars) | 8631.2(2718.9 to 19182.4) | 9684.4(2398.4 to 24240.2) | 0.012 |
| Alcohol use |  |  | 0.043 |
| None | 1569(65.9) | 4638(68.7) |  |
| Moderate | 772(32.4) | 2018(29.9) |  |
| Heavy | 39(1.6) | 96(1.4) |  |
| Smoking status |  |  | <0.001 |
| Never smoked | 1461(58.0) | 4178(58.3) |  |
| Former smoker | 179(7.1) | 731(10.2) |  |
| Current smoker | 880(34.9) | 2255(31.5) |  |
| BMI | 23.9(3.7) | 24.8(41.2) | 0.294 |
| Self-report of Health Status |  |  | 0.132 |
| Excellent | 34(1.3) | 133(1.9) |  |
| Very good | 350(13.9) | 1080(15.1) |  |
| Good | 1361(54.0) | 3758(52.5) |  |
| Fair | 552(21.9) | 1614(22.5) |  |
| Poor | 222(8.8) | 576(8.0) |  |
| ADL limitation | 169(6.8) | 612(8.7) | 0.003 |
| Number of self-reported diagnosed health problems |  |  | 0.003 |
| 0 | 1250(50.5) | 3286(46.7) |  |
| 1 | 804(32.5) | 2371(33.7) |  |
| 2 | 314(12.7) | 988(14.0) |  |
| ≥3 | 107(4.3) | 389(5.5) |  |
| Negative wealth shocks | 351(13.9) | 110(17.2) | 0.043 |

Data are mean (SD), n (%), or median (IQR).

Abbreviations: BMI, body mass index, calculated as weight in kilograms divided by height in meters squared; ADL, activities of daily living; IQR, interquartile range.

**Supplementary Table 5. Comparison of characteristics between the included and excluded participants in Mexico**

|  | Included(n=5024) | Excluded(n=4215) | *P* value |
| --- | --- | --- | --- |
| Mean age(years) | 62.4(7.8) | 66.8(10.3) | <0.001 |
| Gender |  |  | <0.001 |
| Male | 2340(46.6) | 2208(52.4) |  |
| Female | 2684(53.4) | 2007(47.6) |  |
| Education |  |  | 0.052 |
| Lower secondary education or below | 4185(83.6) | 3427(81.8) |  |
| Upper secondary | 200(4.0) | 174(4.2) |  |
| Higher than upper secondary | 622(12.4) | 591(14.1) |  |
| Marital status |  |  | <0.001 |
| Married or living with partner | 3840(76.4) | 2921(69.3) |  |
| Separated or divorced | 349(6.9) | 309(7.3) |  |
| Widowed | 604(12.0) | 784(18.6) |  |
| Never married | 231(4.6) | 201(4.8) |  |
| Minority group | 1569(31.2) | 1041(24.7) | <0.001 |
| Median household size  (people) | 4(3 to 6) | 4(2 to 5) | <0.001 |
| Wealth per capital (2014 US dollars) | 32826.4(12490.9 to 72467.0) | 32499.3(10833.1 to 72434.7) | 0.128 |
| Alcohol use |  |  | 0.001 |
| None | 4004(80.0) | 3488(83.0) |  |
| Moderate | 477(9.5) | 347(8.3) |  |
| Heavy | 524(10.5) | 366(8.7) |  |
| Smoking status |  |  | 0.004 |
| Never smoked | 3102(61.8) | 2491(59.1) |  |
| Former smoker | 1282(25.5) | 1204(28.6) |  |
| Current smoker | 639(12.7) | 518(12.3) |  |
| BMI | 27.7(4.5) | 27.2(4.9) | <0.001 |
| Self-report of Health Status |  |  | <0.001 |
| Excellent | 242(4.8) | 338(8.0) |  |
| Very good | 2449(48.8) | 1981(47.0) |  |
| Good | 1894(37.7) | 1480(35.1) |  |
| Fair | 284(5.7) | 264(6.3) |  |
| Poor | 154(3.1) | 152(3.6) |  |
| ADL limitation | 340(6.8) | 491(11.7) | <0.001 |
| Number of self-reported diagnosed health problems |  |  | <0.001 |
| 0 | 1970(39.5) | 1485(35.4) |  |
| 1 | 1754(35.1) | 1330(31.7) |  |
| 2 | 946(19.0) | 913(21.8) |  |
| ≥3 | 321(6.4) | 469(11.2) |  |
| Negative wealth shocks | 775(15.4) | 533(16.9) | 0.087 |

Data are mean (SD), n (%), or median (IQR).

Abbreviations: BMI, body mass index, calculated as weight in kilograms divided by height in meters squared; ADL, activities of daily living; IQR, interquartile range.

**Supplementary Table 6. Characteristics of the participants in the USA according to the trajectories of depressive symptoms**

| Characteristic | Low trajectory  (n=6398) | Mild trajectory  (n=1517) | Increasing-decreasing trajectory  (n=698) | Decreasing-increasing trajectory  (n=906) |
| --- | --- | --- | --- | --- |
| Mean age(years) | 65.2(9.1) | 65.7(9.4) | 64.7(9.6) | 65.4(9.4) |
| Gender |  |  |  |  |
| Male | 2900(45.3) | 580(38.2) | 233(33.4) | 321(35.4) |
| Female | 3498(54.7) | 937(61.8) | 465(66.6) | 585(64.6) |
| Education |  |  |  |  |
| Lower secondary education or below | 678(10.6) | 289(19.1) | 109(15.6) | 170(18.8) |
| Upper secondary | 2001(31.3) | 547(36.1) | 246(35.2) | 305(33.7) |
| Higher than upper secondary | 3719(58.1) | 681(44.9) | 343(49.1) | 431(47.6) |
| Marital status |  |  |  |  |
| Married or living with partner | 4625(72.3) | 974(64.2) | 455(65.2) | 620(68.4) |
| Separated or divorced | 756(11.8) | 244(16.1) | 112(16.0) | 119(13.1) |
| Widowed | 767(12.0) | 207(13.6) | 86(12.3) | 123(13.6) |
| Never married | 250(3.9) | 92(6.1) | 45(6.4) | 44(4.9) |
| Minority group | 1544(24.1) | 476(31.4) | 237(34.0) | 270(29.8) |
| Median household size  (people) | 2(2 to 3) | 2(2 to 3) | 2(2 to 3) | 2(2 to 3) |
| Wealth per capital (2014 US dollars) | 164984.3(44683.3 to 433083.9) | 90474.0(10517.8 to 320121.2) | 74966.6(8936.7 to 224447.4) | 83351.5(10999.0 to 275317.6) |
| Alcohol use |  |  |  |  |
| None | 3531(55.2) | 900(59.3) | 428(61.3) | 552(60.9) |
| Moderate | 2273(35.5) | 462(30.5) | 197(28.2) | 266(29.4) |
| Heavy | 65949.3) | 155(10.2) | 73(10.5) | 88(9.7) |
| Smoking status |  |  |  |  |
| Never smoked | 3096(48.4) | 670(44.2) | 293(42.0) | 389(43.0) |
| Former smoker | 2699(42.2) | 637(42.0) | 271(38.8) | 361(39.9) |
| Current smoker | 603(9.4) | 210(13.9) | 134(19.2) | 156(17.2) |
| BMI | 28.5(5.6) | 29.2(6.1) | 28.9(6.4) | 28.8(6.0) |
| Self-report of Health Status |  |  |  |  |
| Excellent | 56(0.9) | 46(3.0) | 35(5.0) | 51(5.6) |
| Very good | 587(9.2) | 314(20.7) | 172(24.7) | 205(22.6) |
| Good | 2029(31.7) | 576(38.0) | 271(38.8) | 313(34.5) |
| Fair | 2758(43.1) | 459(30.3) | 179(25.7) | 260(28.7) |
| Poor | 968(15.1) | 122(8.0) | 41(5.9) | 77(8.5) |
| ADL limitation | 290(4.5) | 187(12.3) | 106(15.1) | 125(13.8) |
| Number of self-reported diagnosed health problems |  |  |  |  |
| 0 | 1439(22.5) | 214(14.1) | 85(12.2) | 124(13.7) |
| 1 | 1968(30.8) | 392(25.8) | 177(25.4) | 239(26.4) |
| 2 | 1651(25.8) | 457(30.1) | 193(27.7) | 254(28.0) |
| ≥3 | 1340(20.9) | 454(29.9) | 243(34.8) | 289(31.9) |
| Negative wealth shocks | 387(6.0) | 138(9.1) | 94(13.5) | 814(89.8) |

Data are mean (SD), n (%), or median (IQR).

Abbreviations: BMI, body mass index, calculated as weight in kilograms divided by height in meters squared; ADL, activities of daily living; IQR, interquartile range.

**Supplementary Table 7. Characteristics of the participants in England according to the trajectories of depressive symptoms**

| Characteristic | Low trajectory  (n=3475) | Mild trajectory  (n=772) | Increasing-decreasing trajectory  (n=280) | Decreasing-increasing trajectory  (n=409) |
| --- | --- | --- | --- | --- |
| Mean age(years) | 64.7(7.9) | 67.2(8.8) | 67.3(9.2) | 67.0(8.8) |
| Gender |  |  |  |  |
| Male | 1765(50.8) | 298(38.6) | 83(29.6) | 140(34.2) |
| Female | 1710(49.2) | 474(61.4) | 197(70.4) | 269(65.8) |
| Education |  |  |  |  |
| Lower secondary education or below | 657(18.9) | 228(29.5) | 80(28.5) | 139(34.0) |
| Upper secondary | 1906(54.8) | 408(52.9) | 151(54.1) | 203(49.7) |
| Higher than upper secondary | 912(26.2) | 136(17.7) | 49(17.4) | 67(16.3) |
| Marital status |  |  |  |  |
| Married or living with partner | 2759(79.4) | 547(70.9) | 197(70.4) | 264(64.5) |
| Separated or divorced | 290(8.3) | 78(10.1) | 31(11.1) | 53(13.0) |
| Widowed | 261(7.5) | 114(14.8) | 36(12.9) | 66(16.1) |
| Never married | 165(4.7) | 33(4.3) | 16(5.7) | 26(6.4) |
| Minority group | 85(2.4) | 24(3.1) | 9(3.2) | 15(3.7) |
| Median household size  (people) | 2(2 to 2) | 2(2 to 2) | 2(2 to 2) | 2(1 to 2) |
| Wealth per capital (2014 US dollars) | 379890.7(220022.7 to 629223.5) | 318834.4(171160.4 to 521896.1) | 235989.9(88930.6 to 445037.0) | 282400.2(133289.5 to 472945.6) |
| Alcohol use |  |  |  |  |
| None | 944(27.2) | 282(36.5) | 114(40.7) | 187(45.6) |
| Moderate | 1470(42.3) | 305(39.5) | 106(37.9) | 139(34.0) |
| Heavy | 1061(30.5) | 185(24.0) | 60(21.4) | 83(20.3) |
| Smoking status |  |  |  |  |
| Never smoked | 1462(42.1) | 300(38.9) | 110(39.4) | 164(40.1) |
| Former smoker | 1731(49.8) | 389(50.4) | 141(50.2) | 192(46.9) |
| Current smoker | 282(8.1) | 83(10.8) | 29(10.4) | 53(13.0) |
| BMI | 27.8(4.7) | 28.6(5.4) | 29.6(5.1) | 29.2(5.3) |
| Self-report of Health Status |  |  |  |  |
| Excellent | 43(1.2) | 32(4.1) | 14(5.0) | 14(3.4) |
| Very good | 335(9.6) | 157(20.3) | 70(25.0) | 106(25.9) |
| Good | 1065(30.6) | 285(36.9) | 122(43.6) | 164(40.1) |
| Fair | 1369(39.4) | 231(29.9) | 54(19.3) | 93(22.7) |
| Poor | 663(19.1) | 67(8.7) | 20(7.1) | 32(7.8) |
| ADL limitation | 222(6.4) | 132(17.1) | 65(23.2) | 75(18.3) |
| Number of self-reported diagnosed health problems |  |  |  |  |
| 0 | 1297(37.3) | 187(24.2) | 73(26.1) | 92(22.5) |
| 1 | 1235(35.5) | 287(37.2) | 78(27.9) | 125(30.6) |
| 2 | 677(19.5) | 188(24.4) | 78(27.9) | 129(31.5) |
| ≥3 | 266(7.7) | 110(14.2) | 51(18.2) | 63(15.4) |
| Negative wealth shocks | 94(2.7) | 30(3.9) | 14(5.0) | 18(4.4) |

Data are mean (SD), n (%), or median (IQR).

Abbreviations: BMI, body mass index, calculated as weight in kilograms divided by height in meters squared; ADL, activities of daily living; IQR, interquartile range.

**Supplementary Table 8. Characteristics of the participants in China according to the trajectories of depressive symptoms**

| Characteristic | Low trajectory  (n=1149) | Mild trajectory  (n=733) | Increasing-decreasing trajectory  (n=310) | Decreasing-increasing trajectory  (n=271) | High trajectory  (n=57) |
| --- | --- | --- | --- | --- | --- |
| Mean age(years) | 56.5(8.1) | 57.3(7.8) | 56.4(7.8) | 57.0(7.5) | 57.7(8.3) |
| Gender |  |  |  |  |  |
| Male | 724(63.0) | 379(51.7) | 140(45.2) | 101(37.3) | 25(43.9) |
| Female | 425(37.0) | 354(48.3) | 170(54.8) | 170(62.7) | 32(56.1) |
| Education |  |  |  |  |  |
| Lower secondary education or below | 949(82.6) | 656(89.5) | 288(92.9) | 248(91.5) | 53(93.0) |
| Upper secondary | 176(15.3) | 73(10.0) | 18(5.8) | 23(8.5) | 4(7.0) |
| Higher than upper secondary | 24(2.1) | 4(0.5) | 4(1.3) | 0(0.0) | 0(0.0) |
| Marital status |  |  |  |  |  |
| Married or living with partner | 1079(93.9) | 673(91.8) | 288(92.9) | 240(88.6) | 50(87.7) |
| Separated or divorced | 11(1.0) | 7(1.0) | 3(1.0) | 0(0.0) | 0(0.0) |
| Widowed | 58(5.0) | 47(6.4) | 16(5.2) | 28(10.3) | 5(8.8) |
| Never married | 1(0.1) | 6(0.8) | 3(1.0) | 3(1.1) | 2(3.5) |
| Minority group | 675(58.7) | 505(68.9) | 229(73.9) | 194(71.6) | 42(73.7) |
| Median household size  (people) | 3(2 to 4) | 3(2 to 5) | 3(2 to 5) | 3(2 to 5) | 3(2 to 4) |
| Wealth per capital (2014 US dollars) | 11172.7(3263.1 to 24135.5) | 8344.2(2884.4 to 17597.5) | 6333.9(2405.4 to 15814.4) | 7220.8(1959.0 to 15394.9) | 4729.9(1606.4 to 11339.3) |
| Alcohol use |  |  |  |  |  |
| None | 698(60.8) | 475(64.9) | 227(73.2) | 202(74.3) | 39(69.2) |
| Moderate | 431(37.5) | 247(33.7) | 77(24.8) | 64(23.7) | 17(28.9) |
| Heavy | 20(1.7) | 11(1.5) | 6(2.0) | 5(2.0) | 1(1.9) |
| Smoking status |  |  |  |  |  |
| Never smoked | 614(53.4) | 426(58.1) | 192(61.9) | 192(70.8) | 37(64.9) |
| Former smoker | 84(7.3) | 58(7.9) | 21(6.8) | 14(5.2) | 2(3.5) |
| Current smoker | 451(39.3) | 249(34.0) | 97(31.3) | 65(24.0) | 18(31.6) |
| BMI | 23.9(3.6) | 23.9(4.0) | 23.8(3.6) | 24.1(3.8) | 24.0(3.2) |
| Self-report of Health Status |  |  |  |  |  |
| Excellent | 5(0.4) | 13(1.8) | 6(1.9) | 6(2.2) | 4(7.0) |
| Very good | 100(8.7) | 117(16.0) | 58(18.7) | 59(21.8) | 16(28.1) |
| Good | 585(50.9) | 424(57.8) | 173(55.8) | 155(57.2) | 25(43.9) |
| Fair | 309(26.9) | 138(18.8) | 58(18.7) | 40(14.8) | 7(12.3) |
| Poor | 150(13.1) | 41(5.6) | 15(4.8) | 11(4.1) | 5(8.8) |
| ADL limitation | 43(3.8) | 59(8.1) | 29(9.4) | 27(10.0) | 11(19.3) |
| Number of self-reported diagnosed health problems |  |  |  |  |  |
| 0 | 655(57.0) | 357(48.7) | 136(43.9) | 110(40.6) | 16(17.2) |
| 1 | 362(31.5) | 244(33.3) | 109(35.1) | 85(31.3) | 21(37.4) |
| 2 | 102(8.9) | 97(13.3) | 46(14.9) | 55(20.3) | 16(28.3) |
| ≥3 | 30(2.6) | 35(4.7) | 19(6.2) | 21(7.8) | 4(7.2) |
| Negative wealth shocks | 124(10.8) | 117(16.0) | 53(17.1) | 44(16.2) | 13(22.8) |

Data are mean (SD), n (%), or median (IQR).

Abbreviations: BMI, body mass index, calculated as weight in kilograms divided by height in meters squared; ADL, activities of daily living; IQR, interquartile range.

**Supplementary Table 9. Characteristics of the participants in Mexico according to the trajectories of depressive symptoms**

| Characteristic | Low trajectory  (n=2175) | Mild trajectory  (n=916) | Increasing-decreasing trajectory  (n=742) | Decreasing-increasing trajectory  (n=879) | Decreasing trajectory  (n=312) |
| --- | --- | --- | --- | --- | --- |
| Mean age(years) | 61.8(7.5) | 62.6(7.9) | 63.0(8.1) | 63.4(7.7) | 62.5(7.5) |
| Gender |  |  |  |  |  |
| Male | 1160(53.3) | 436(47.6) | 276(37.2) | 335(38.1) | 133(42.6) |
| Female | 1015(46.7) | 480(52.4) | 466(62.8) | 544(61.9) | 179(57.4) |
| Education |  |  |  |  |  |
| Lower secondary education or below | 1673(76.9) | 775(84.6) | 652(87.9) | 813(92.5) | 287(91.8) |
| Upper secondary | 113(5.2) | 35(3.8) | 26(3.5) | 18(2.1) | 8(2.6) |
| Higher than upper secondary | 389(17.9) | 106(11.6) | 64(8.6) | 48(5.4) | 17(5.5) |
| Marital status |  |  |  |  |  |
| Married or living with partner | 1665(76.6) | 692(75.5) | 571(77.0) | 671(76.3) | 241(77.2) |
| Separated or divorced | 158(7.3) | 59(6.4) | 45(6.1) | 62(7.1) | 25(8.0) |
| Widowed | 236(10.9) | 116(12.7) | 97(13.1) | 118(13.4) | 37(11.9) |
| Never married | 116(5.3) | 49(5.3) | 29(3.9) | 28(3.2) | 9(2.9) |
| Minority group | 602(27.7) | 318(34.7) | 239(32.2) | 293(33.3) | 117(37.5) |
| Median household size  (people) | 4(3 to 6) | 4(3 to 6) | 4(3 to 6) | 4(3 to 6) | 4(3 to 6) |
| Wealth per capital (2014 US dollars) | 38433.4(16385.1 to 83956.4) | 31382.5(12185.7 to 69753.8) | 27569.5(10219.0 to 67456.2) | 29054.9(9200.2 to 59311.2) | 27296.4(11335.2 to 45389.9) |
| Alcohol use |  |  |  |  |  |
| None | 1670(76.8) | 703(76.8) | 632(85.1) | 752(85.6) | 258(82.7) |
| Moderate | 250(11.5) | 92(10.0) | 46(6.2) | 69(7.8) | 24(7.7) |
| Heavy | 255(11.7) | 121(13.2) | 64(8.6) | 58(6.6) | 30(9.6) |
| Smoking status |  |  |  |  |  |
| Never smoked | 1271(58.4) | 553(60.4) | 488(65.8) | 594(67.6) | 197(63.1) |
| Former smoker | 596(27.4) | 236(25.8) | 170(22.9) | 198(22.5) | 82(26.3) |
| Current smoker | 308(14.2) | 127(13.9) | 84(11.3) | 87(9.9) | 33(10.6) |
| BMI | 27.4(4.4) | 27.9(4.5) | 27.8(4.7) | 27.9(4.7) | 28.0(4.8) |
| Self-report of Health Status |  |  |  |  |  |
| Excellent | 53(2.4) | 48(5.2) | 53(7.1) | 61(6.9) | 27(8.7) |
| Very good | 871(40.1) | 485(52.9) | 417(56.2) | 497(56.5) | 179(57.4) |
| Good | 979(45.0) | 322(35.2) | 219(29.5) | 284(32.3) | 91(29.2) |
| Fair | 163(7.5) | 42(4.6) | 40(5.4) | 27(3.1) | 12(3.8) |
| Poor | 109(5.0) | 19(2.1) | 13(1.8) | 10(1.1) | 3(1.0) |
| ADL limitation | 78(3.6) | 64(7.0) | 68(9.1) | 103(11.8) | 28(9.1) |
| Number of self-reported diagnosed health problems |  |  |  |  |  |
| 0 | 1053(48.4) | 321(35.1) | 237(31.9) | 272(30.9) | 104(33.4) |
| 1 | 723(33.2) | 345(37.6) | 268(36.1) | 315(35.9) | 115(36.9) |
| 2 | 310(14.3) | 197(21.5) | 172(23.2) | 202(23.0) | 69(21.9) |
| ≥3 | 89(4.1) | 53(5.8) | 65(8.8) | 90(10.3) | 24(7.7) |
| Negative wealth shocks | 318(14.6) | 149(16.3) | 109(14.7) | 146(16.6) | 53(17.0) |

Data are mean (SD), n (%), or median (IQR).

Abbreviations: BMI, body mass index, calculated as weight in kilograms divided by height in meters squared; ADL, activities of daily living; IQR, interquartile range.

**Supplementary Table 10. Associations of negative wealth shocks with subsequent positive for depressive symptoms—results of additional analysis**

|  | HRS  (the USA) | ELSA  (England) | CHARLS  (China) | MHAS  (Mexico) |
| --- | --- | --- | --- | --- |
| **Main analysis** | | | | |
| Participants, No. | 9519 | 4936 | 2520 | 5024 |
| OR (95% CI) | 1.73(1.40-2.16) | 1.71(1.09-2.70) | 1.38(1.09-1.75) | 1.06(0.86-1.29) |
| Interaction P-value | Reference | 0.565 | 0.498 | 0.006 |
| **Age subgroup analysis** | | | | |
| ＜65 | 2.13(1.59-2.84) | 2.90(1.50-5.62) | 1.46(1.11-1.91) | 1.08(0.82-1.41) |
| ≥65 | 1.35(0.94-1.92) | 1.03(0.53-2.02) | 1.36(0.82-2.27) | 1.00(0.73-1.37) |
| **Gender subgroup analysis** | | | | |
| Male | 1.91(1.30-2.80) | 1.77(0.80-3.92) | 1.43(1.04-1.99) | 1.06(0.76-1.47) |
| Female | 1.60(1.21-2.11) | 1.51(0.87-2.65) | 1.33(0.94-1.88) | 1.02(0.79-1.32) |
| **Only individuals included with complete covariates** | | | | |
| Participants, No. | 8757 | 3463 | 1184 | 4412 |
| OR (95% CI) | 1.71(1.26-2.33) | 1.75(0.76-4.06) | 1.53(1.06-2.19) | 0.98(0.77-1.25) |
| Interaction P-value | Reference | 0.966 | 0.781 | 0.011 |
| **Excluding individuals who did not experience negative wealth shocks in the second wave but did so afterwards** | | | | |
| Participants, No. | 8336 | 4698 | 2021 | 3969 |
| OR (95% CI) | 1.90(1.50-2.42) | 1.67(1.02-2.75) | 1.51(1.16-1.98) | 1.15(0.92-1.44) |
| Interaction P-value | Reference | 0.606 | 0.249 | 0.013 |
| **Additional adjustment for labor force participation** | | | | |
| OR (95% CI) | 1.74(1.40-2.17) | 1.71(1.09-2.69) | 1.38(1.09-1.75) | 1.06(0.86-1.29) |
| Interaction P-value | Reference | 0.580 | 0.478 | 0.005 |
| **Additional adjustment for pension receipt** | | | | |
| OR (95% CI) | 1.72(1.39-2.14) | 1.72(1.09-2.69) | 1.37(1.08-1.75) | 1.06(0.86-1.29) |
| Interaction P-value | Reference | 0.480 | 0.600 | 0.011 |
| **Additional adjustment for labor force participation and pension receipt** | | | | |
| OR (95% CI) | 1.73(1.39-2.15) | 1.72(1.09-2.71) | 1.38(1.09-1.75) | 1.06(0.86-1.29) |
| Interaction P-value | Reference | 0.487 | 0.583 | 0.008 |
| **Excluding the final wave** | | | | |
| OR (95% CI) | 2.03(1.53-2.68) | 1.99(1.11-3.56) | 1.55(1.16-2.08) | 1.06(0.86-1.29) |
| Interaction P-value | Reference | 0.652 | 0.646 | 0.022 |
| **50% threshold for negative wealth shocks** | | | | |
| OR (95% CI) | 1.48(1.25-1.75) | 1.57(1.15-2.15) | 1.37(1.11-1.67) | 0.98(0.83-1.17) |
| Interaction P-value | Reference | 0.308 | 0.907 | 0.003 |

**Supplementary Figure 5. Trajectories of depressive symptoms after baseline**—**only individuals included with complete covariates, by country**

**
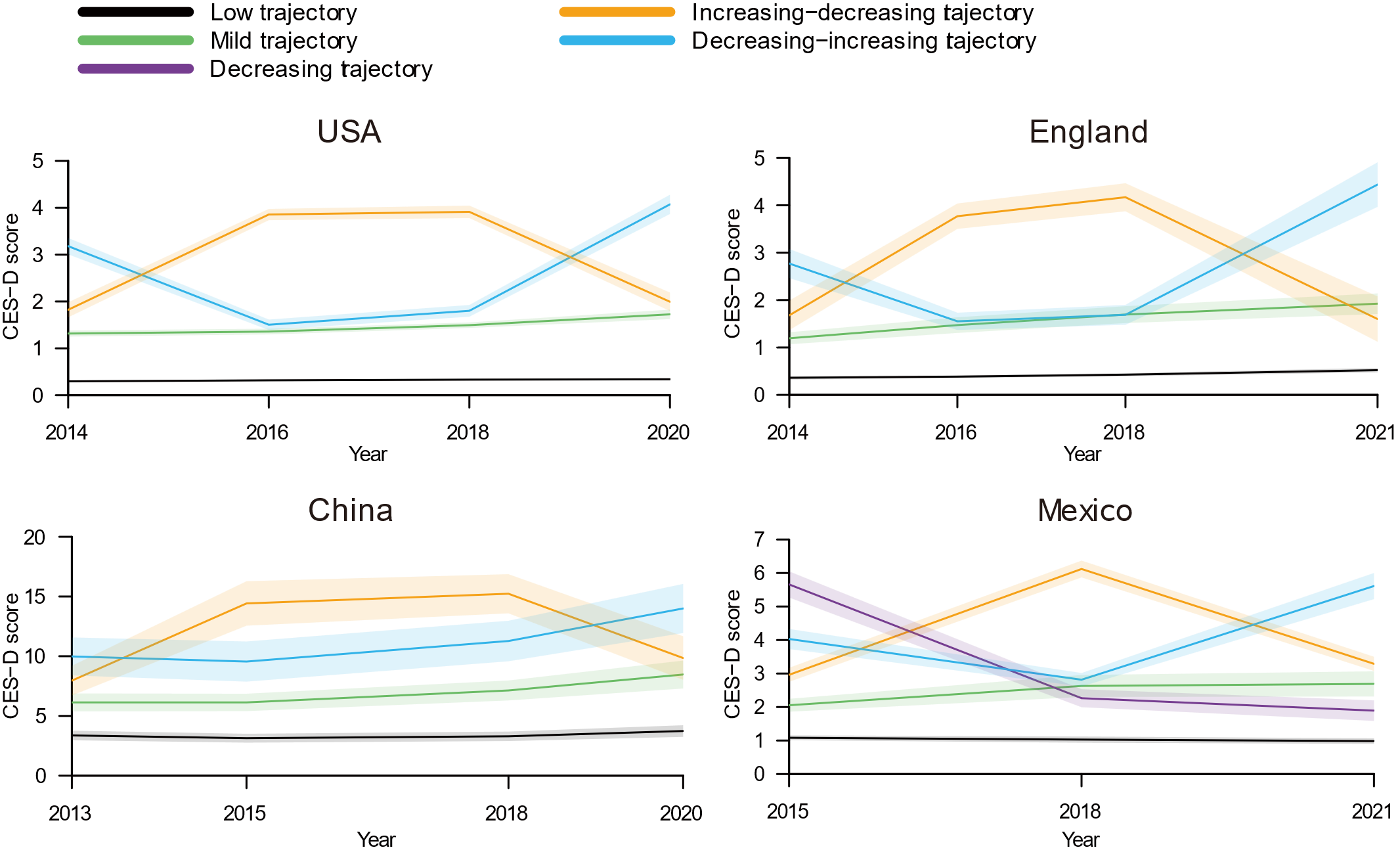
**

**Supplementary Table 11. Associations of negative wealth shocks with trajectories of depressive symptoms** —**only individuals included with complete covariates, by country**

| Country | Trajectory | Participants, No. | Negative wealth shock |
| --- | --- | --- | --- |
| HRS (the USA) | Low | 5906 | Reference |
|  | Mild | 1394 | 1.43(1.13-1.80) |
|  | Increasing-decreasing | 641 | 1.95(1.46-2.60) |
|  | Decreasing-increasing | 816 | 1.49(1.12-1.98) |
| ELSA(England) | Low | 2485 | Reference |
|  | Mild | 515 | 1.20(0.59-2.47) |
|  | Increasing-decreasing | 174 | 3.36(1.57-7.16) |
|  | Decreasing-increasing | 289 | 1.92(0.91-4.10) |
| CHARLS(China) | Low | 525 | Reference |
|  | Mild | 376 | 1.59(1.09-2.34) |
|  | Increasing-decreasing | 103 | 2.11(1.22-3.66) |
|  | Decreasing-increasing | 180 | 1.28(0.77-2.13) |
| MHAS(Mexico) | Low | 1900 | Reference |
|  | Mild | 777 | 1.15(0.91-1.44) |
|  | Increasing-decreasing | 668 | 0.95(0.74-1.22) |
|  | Decreasing-increasing | 750 | 1.16(0.92-1.46) |
|  | Decreasing | 317 | 0.96(0.68-1.34) |

**Supplementary Figure 6. Trajectories of depressive symptoms after baseline**—**excluding individuals who did not experience negative wealth shocks in the second wave but did so afterwards, by country**

**
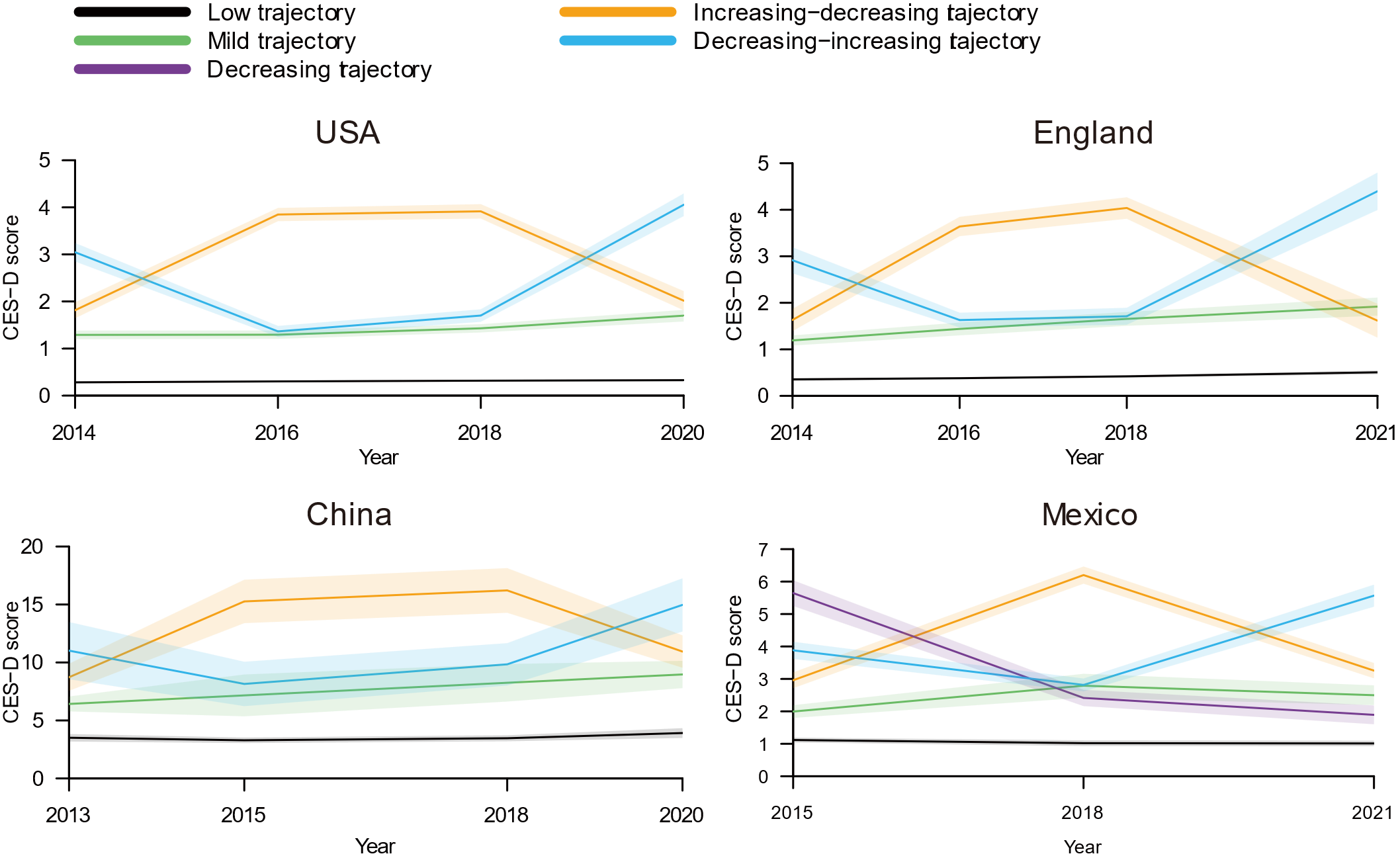
**

**Supplementary Table 12. Associations of negative wealth shocks with trajectories of depressive symptoms** —**excluding individuals who did not experience negative wealth shocks in the second wave but did so afterwards, by country**

| Country | Trajectory | Participants, No. | Negative wealth shock |
| --- | --- | --- | --- |
| HRS (the USA) | Low | 5656 | Reference |
|  | Mild | 1323 | 1.54(1.25-1.88) |
|  | Increasing-decreasing | 597 | 2.28(1.88-2.94) |
|  | Decreasing-increasing | 760 | 1.35(1.04-1.77) |
| ELSA(England) | Low | 3301 | Reference |
|  | Mild | 752 | 1.17(0.67-2.05) |
|  | Increasing-decreasing | 260 | 2.01(0.99-4.06) |
|  | Decreasing-increasing | 385 | 1.50(0.77-2.93) |
| CHARLS(China) | Low | 1015 | Reference |
|  | Mild | 650 | 1.53(1.15-2.05) |
|  | Increasing-decreasing | 153 | 1.82(1.18-2.79) |
|  | Decreasing-increasing | 203 | 1.91(1.30-2.82) |
| MHAS(Mexico) | Low | 1765 | Reference |
|  | Mild | 624 | 1.39(1.14-1.71) |
|  | Increasing-decreasing | 535 | 1.36(1.10-1.69) |
|  | Decreasing-increasing | 728 | 1.37(1.13-1.66) |
|  | Decreasing | 317 | 1.12(0.85-1.49) |

**Supplementary Table 13. Associations of negative wealth shocks with trajectories of depressive symptoms** —**additional adjustment for labor force participation, by country**

|  | Trajectory | Participants, No. | Odds Ratio (95% CI) |
| --- | --- | --- | --- |
| HRS (the USA) | Low | 6398 | Reference |
|  | Mild | 1517 | 1.38(1.11-1.73) |
|  | Increasing-decreasing | 698 | 2.02(1.54-2.63) |
|  | Decreasing-increasing | 906 | 1.49(1.14-1.94) |
| ELSA(England) | Low | 3475 | Reference |
|  | Mild | 772 | 1.15(0.68-1.95) |
|  | Increasing-decreasing | 280 | 2.10(1.12-3.94) |
|  | Decreasing-increasing | 409 | 1.46(0.79-2.71) |
| CHARLS(China) | Low | 1149 | Reference |
|  | Mild | 733 | 1.52(1.16-2.00) |
|  | Increasing-decreasing | 310 | 1.57(1.09-2.25) |
|  | Decreasing-increasing | 271 | 1.37(0.91-2.05) |
|  | High | 57 | 2.47(1.28-4.77) |
| MHAS(Mexico) | Low | 2175 | Reference |
|  | Mild | 916 | 1.15(0.93-1.41) |
|  | Increasing-decreasing | 742 | 1.01(0.80-1.27) |
|  | Decreasing-increasing | 879 | 1.18(0.96-1.46) |
|  | Decreasing | 312 | 1.25(0.92-1.71) |

**Supplementary Table 14. Associations of negative wealth shocks with trajectories of depressive symptoms** —a**dditional adjustment for pension receipt, by country**

|  | Trajectory | Participants, No. | Odds Ratio (95% CI) |
| --- | --- | --- | --- |
| HRS (the USA) | Low | 6398 | Reference |
|  | Mild | 1517 | 1.43(1.15-1.79) |
|  | Increasing-decreasing | 698 | 1.97(1.50-2.59) |
|  | Decreasing-increasing | 906 | 1.46(1.11-1.91) |
| ELSA(England) | Low | 3475 | Reference |
|  | Mild | 772 | 1.18(0.70-1.99) |
|  | Increasing-decreasing | 280 | 2.13(1.14-3.97) |
|  | Decreasing-increasing | 409 | 1.51(0.83-2.77) |
| CHARLS(China) | Low | 1149 | Reference |
|  | Mild | 733 | 1.53(1.16-2.01) |
|  | Increasing-decreasing | 310 | 1.52(1.06-2.18) |
|  | Decreasing-increasing | 271 | 1.39(0.93-2.08) |
|  | High | 57 | 2.42(1.26-4.69) |
| MHAS(Mexico) | Low | 2175 | Reference |
|  | Mild | 916 | 1.15(0.93-1.42) |
|  | Increasing-decreasing | 742 | 1.01(0.80-1.27) |
|  | Decreasing-increasing | 879 | 1.19(0.96-1.47) |
|  | Decreasing | 312 | 1.26(0.92-1.72) |

**Supplementary Table 15. Associations of negative wealth shocks with trajectories of depressive symptoms** —**additional adjustment for labor force participation and pension receipt, by country**

|  | Trajectory | Participants, No. | Odds Ratio (95% CI) |
| --- | --- | --- | --- |
| HRS (the USA) | Low | 6398 | Reference |
|  | Mild | 1517 | 1.42(1.14-1.77) |
|  | Increasing-decreasing | 698 | 1.98(1.51-2.59) |
|  | Decreasing-increasing | 906 | 1.47(1.12-1.92) |
| ELSA(England) | Low | 3475 | Reference |
|  | Mild | 772 | 1.15(0.68-1.96) |
|  | Increasing-decreasing | 280 | 2.16(1.16-4.03) |
|  | Decreasing-increasing | 409 | 1.51(0.82-2.78) |
| CHARLS(China) | Low | 1149 | Reference |
|  | Mild | 733 | 1.53(1.16-2.01) |
|  | Increasing-decreasing | 310 | 1.55(1.17-2.03) |
|  | Decreasing-increasing | 271 | 1.40(0.93-2.10) |
|  | High | 57 | 2.44(1.26-4.73) |
| MHAS(Mexico) | Low | 2175 | Reference |
|  | Mild | 916 | 1.15(0.93-1.41) |
|  | Increasing-decreasing | 742 | 1.00(0.80-1.27) |
|  | Decreasing-increasing | 879 | 1.19(0.96-1.47) |
|  | Decreasing | 312 | 1.26(0.92-1.71) |

**Supplementary Table 16. Associations of negative wealth shocks with trajectories of depressive symptoms** —**50% threshold for negative wealth shocks, by country**

|  | Trajectory | Participants, No. | Odds Ratio (95% CI) |
| --- | --- | --- | --- |
| HRS (the USA) | Low | 6398 | Reference |
|  | Mild | 1517 | 1.20(1.02-1.41) |
|  | Increasing-decreasing | 698 | 1.71(1.40-2.10) |
|  | Decreasing-increasing | 906 | 1.26(1.03-1.53) |
| ELSA(England) | Low | 3475 | Reference |
|  | Mild | 772 | 1.05(0.76-1.45) |
|  | Increasing-decreasing | 280 | 2.07(1.41-3.04) |
|  | Decreasing-increasing | 409 | 1.45(0.99-2.11) |
| CHARLS(China) | Low | 1149 | Reference |
|  | Mild | 733 | 1.240.99-1.55) |
|  | Increasing-decreasing | 310 | 1.34(0.99-1.81) |
|  | Decreasing-increasing | 271 | 1.24(0.90-1.70) |
|  | High | 57 | 2.40(1.38-4.18) |
| MHAS(Mexico) | Low | 2175 | Reference |
|  | Mild | 916 | 1.07(0.90-1.26) |
|  | Increasing-decreasing | 742 | 0.92(0.77-1.11) |
|  | Decreasing-increasing | 879 | 1.17(0.99-1.39) |
|  | Decreasing | 312 | 1.03(0.79-1.33) |
